# Outcomes of high-dose oral beta-lactam definitive therapy compared to fluoroquinolone or trimethoprim-sulfamethoxazole oral therapy for bacteremia secondary to a urinary tract infection

**DOI:** 10.1017/ash.2023.435

**Published:** 2023-09-08

**Authors:** Abigail C. Geyer, Kali M. VanLangen, Andrew P. Jameson, Lisa E. Dumkow

**Affiliations:** 1 Department of Pharmacy, Trinity Health Grand Rapids, Grand Rapids, MI, USA; 2 Ferris State University, College of Pharmacy, Grand Rapids, MI, USA; 3 Division of Infectious Disease, Trinity Health Grand Rapids, Grand Rapids, MI, USA; 4 Department of Medicine, Michigan State College of Human Medicine, Grand Rapids, MI, USA

**Keywords:** Oral beta-lactams, bloodstream infections, dose-optimization, adherence optimization, urinary tract infection

## Abstract

**Objective:**

Compare outcomes of patients receiving high-dose oral beta-lactam versus standard oral therapy for Enterobacterales bacteremia from a urinary tract infection (UTI).

**Design:**

Retrospective, multicenter, observational cohort.

**Setting:**

Three Michigan community teaching hospitals.

**Patients:**

Adult patients admitted between February 1, 2020, and October 1, 2022, with gram-negative bacteremia from a urinary source were evaluated. Patients receiving active empiric intravenous (IV) antibiotics and transitioned to appropriately dosed oral cephalexin, amoxicillin, fluoroquinolone (FQ), or trimethoprim/sulfamethoxazole (TMP/SMX) were included. Patients receiving less than 72 hours of oral therapy, diagnosed with renal abscess, lobar nephronia, or expired during admission were excluded.

**Methods:**

Standard oral therapy was defined as FQ or TMP/SMX. The primary outcome compared the composite of recurrent bacteremia or mortality within 30 days of therapy between groups. Secondary outcomes compared recurrent UTI, emergency department or hospital readmission, and *Clostridioides difficile* within 30 days.

**Results:**

194 patients were included (beta-lactam, *n* = 75 vs standard therapy, *n* =119). Patients in both groups were treated for a median of 11 days, with 4 days IV and 7 days oral therapy. There was no difference in the primary outcome between groups (beta-lactam 1.3% vs standard therapy 1.7%, OR 1.27 [95% CI 0.11–14.2]). No patients experienced *C. difficile* in either group (*p* = 1.0). Infectious disease consultation was independently associated with standard therapy prescribing (OR 4.4 [95% CI 2.24–8.26]).

**Conclusion:**

High-dose oral beta-lactams were as safe and effective as oral FQ or TMP/SMX for the treatment of bacteremia from a urinary source. Most patients received 8–10 days of therapy in both groups.

## Background

Complicated urinary tract infections (UTI) and pyelonephritis are often treated with intravenous (IV) antibiotics followed by definitive therapy with an oral fluoroquinolone (FQ) or trimethoprim/sulfamethoxazole (TMP/SMX) given these agents’ high oral bioavailability and ability to obtain serum concentrations similar to IV administration.^1^ These agents have historically been preferred for definitive treatment of pyelonephritis and bacteremia; however, increasing resistance and risk of adverse drug events may limit their utility. Oral beta-lactams as definitive therapy for Enterobacterales bacteremia secondary to a urinary source are less widely used as they have been thought to be less effective due to concern of subtherapeutic serum concentrations.^2,3^ Recent literature suggests oral beta-lactams are potential options for definitive therapy in these cases; however, these studies included a variety of oral beta-lactams and dosing strategies.^4–7^

There is currently no preferred beta-lactam agent or standard dosing recommendations for beta-lactams for treating gram-negative bacteremia from a urinary source. A recent consensus statement from a small group of infectious diseases specialists published in 2021 provides recommendations regarding the use of beta-lactams for uncomplicated Enterobacterales bacteremia from a variety of infectious sources.^8^ Oral fluoroquinolones and TMP/SMX were not always the selected oral agent; instead, providers chose agents based on patient-specific factors including risk for toxicity. Oral beta-lactams with high oral bioavailability, low protein binding, and high rates of urinary excretion were considered reasonable options after receiving multiple days of IV therapy (Supplementary Table 1).^9–16^ Local practices of dosing beta-lactams for Enterobacterales bacteremia from a urinary source may also differ from the regimens utilized in these retrospective cohort studies and consensus statement. The purpose of this study was to evaluate the outcomes of utilizing high-dose cephalexin 1 g three times daily or amoxicillin 1 g three times daily as definitive oral therapy for Enterobacterales bacteremia from a urinary source compared to optimally dosed alternative therapy.

## Methods

### Study design, site, and patient selection

This was a multicenter, retrospective cohort study of patients with Enterobacterales bacteremia due to a urinary source treated at three hospitals in Michigan within a large health system. These institutions were selected due to similarities of their antibiograms for uropathogens, antimicrobial stewardship program ages and practices, and dosing recommendations for oral (PO) treatment of bacteremia. Data were collected for patients treated between February 1, 2020, and October 1, 2022. Patients were included in the beta-lactam or standard therapy cohort based on definitive oral agent utilized. The beta-lactam group included patients who received high-dose amoxicillin or cephalexin, while the standard treatment included ciprofloxacin, levofloxacin, or TMP/SMX. This study was approved by the institutional review board at this health system.

Adults 18 years or older with matching urine and blood cultures with *Escherichia coli, Klebsiella pneumoniae or oxyctoca, or Proteus mirabilis* who received an empiric antibiotic regimen active against the isolated pathogen and then transitioned to appropriately dosed oral therapy (Supplementary Table 2) were included. Patients were excluded who received less than 72 hours of oral therapy, were pregnant, had a history of renal transplant, were treated for renal abscess or had lobar nephronia, who expired during hospital admission, or discharged with hospice. All patients meeting inclusion criteria during the study timeframe were evaluated.

### Data collection and study endpoints

All data were collected from Epic Care Everywhere (Epic Systems Corporation, Verona, WI) which provides access to both inpatient and outpatient records. The primary outcome compared the composite of mortality or recurrent bacteremia within 30 days of completing high-dose oral beta-lactam versus standard oral therapy. Recurrent bacteremia was defined as a positive blood culture of the same genus and species as the initial blood culture from a urinary source. Secondary outcomes compared between groups included incidence of recurrent UTI treatment, readmission to the emergency department (ED) or hospital, and C*lostridioides difficile* infection within 30 days of oral therapy completion. Treatment of recurrent UTI was defined as a positive urine culture that received antibiotic treatment in either the inpatient or outpatient setting. Recurrent UTI was further classified as symptomatic versus asymptomatic.

### Statistical analysis

Statistical analysis was performed using SPSS v.22 (IBM, SPSS Inc., Armonk, NY). Categorical variables were analyzed using Pearson chi-square or Fisher’s exact test. Continuous variables were analyzed using Mann-Whitney test. Statistical significance was set as a p-value of less than 0.05. Independent risk factors for receiving standard therapy vs. beta-lactam therapy were modeled using logistic regression and expressed as odds ratios and 95% confidence intervals. A bivariate analysis was conducted to identify variables with *p*-value of ≤0.2 to include in the logistic regression model.

## Results

### Study population

**Figure 1.**
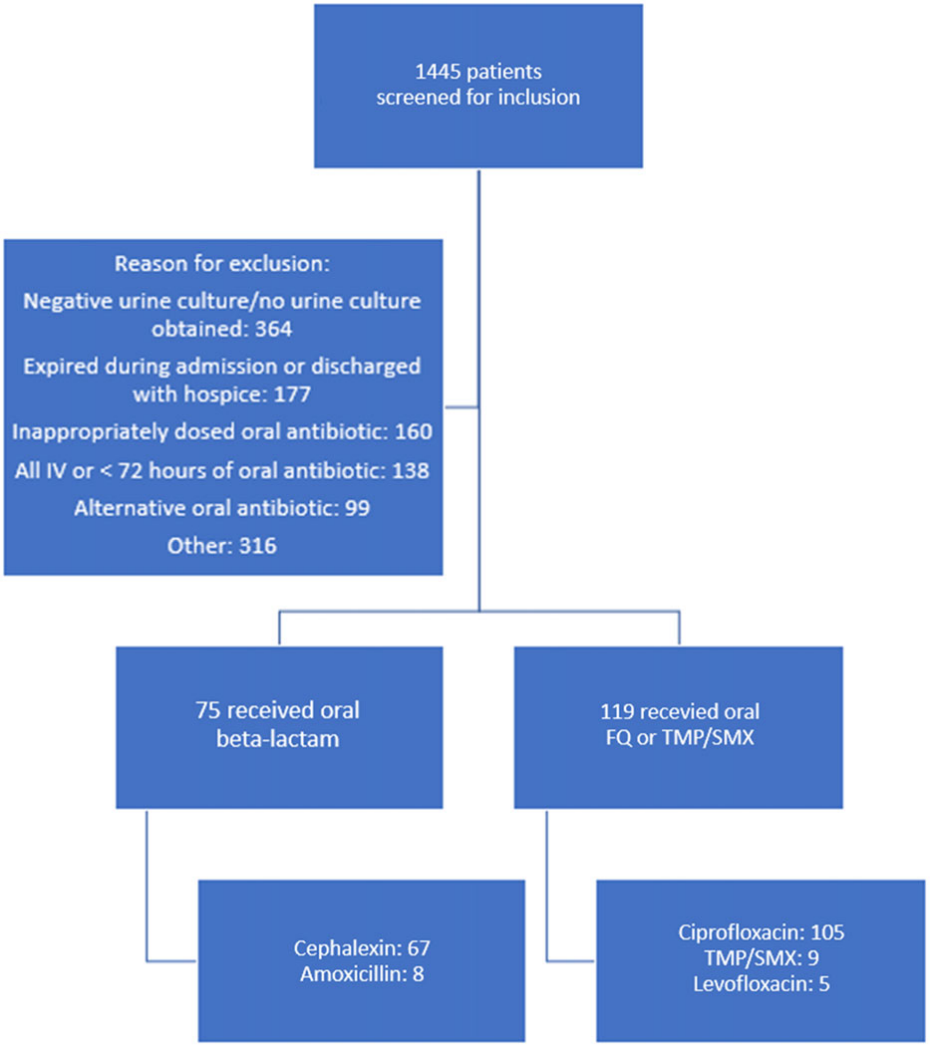
Diagram of inclusion, exclusion, and exposures.

A total of 1445 patients were screened for inclusion with 194 patients meeting eligibility (Figure 1). Seventy-five patients received a high-dose oral beta-lactam for definitive therapy, while 119 patients received appropriately dosed standard oral therapy (Figure 1). Baseline characteristics are presented in Table 1. Patients who received standard oral treatment were more likely to have been admitted to the intensive care unit (ICU), have a Foley catheter inserted during hospital admission, have an infectious disease consult, and have repeat blood cultures ordered compared to the beta-lactam group. All other characteristics were similar between the two treatment groups. The most commonly isolated pathogen was *E. coli* (77.3%). Susceptibilities of the isolated pathogens to first-generation cephalosporins and fluoroquinolones were over 90% in both groups. Patients received a median of 4 days of IV antibiotic therapy prior to oral switch in both groups (beta-lactam: 4 days [IQR] 3–5 days vs. standard therapy: 4 days [IQR] 3–5 days, *p* = 0.205) and a median of 7 days of oral therapy (beta-lactam: 7 days [IQR] 6–10 vs. standard therapy: 7 days [IQR] 5–10; *p* = 0.332). Duration of antibiotic treatment was the same in both groups (median 11 days; [IQR] 10–14 days; *p* = 0.802). The majority of patients in both groups were treated for a total of 8-10 days. Hospital length of stay was the same in both groups (median 5 days; [IQR] 4–6 days; *p* = 0.330).

**Table 1:** . Baseline characteristics

Characteristic	Beta-lactam (*n* = 75)	FQ or TMP/SMX (*n* = 119)	*p*-value
Female sex, *n* (%)	49 (65.3)	76 (63.9)	0.835
Age, median (IQR)	71 (60–79)	72 (62–79)	0.730
BMI ≥35 kg/m^2^, *n* (%)	14 (18.9)	25 (21)	0.725
Allergy, *n* (%)			
• Penicillin	13 (17.3)	20 (16.8)	0.924
• Cephalosporin	4 (5.3)	7 (5.9)	1.0
• FQ	4 (5.3)	3 (2.5)	0.433
• Sulfa	17 (22.7)	18 (15.1)	0.183
Charlson comorbidity index, median (IQR)	1 (0–2)	2 (1–3)	0.213
Renal stone, *n* (%)	16 (21.3)	35 (29.5)	0.213
Creatinine clearance at transition to PO antibiotic, *n* (%)			
• ≥50 mL/min	43 (57.3)	45 (38.1)	0.034
• 30–49 mL/min	22 (29.3)	42 (35.6)	
• 10–29 mL/min	10 (13.3)	28 (23.7)	
• <10 mL/min or dialysis	0 (0)	3 (2.5)	
Urologic procedure, *n* (%)	15 (20)	30 (25.2)	0.402
Foley catheter status, *n* (%)			
• Present on admission	6 (8)	13 (10.9)	0.505
• Present during admission	20 (26.7)	53 (44.5)	0.012
• Present on discharge	7 (9.3)	20 (16.8)	0.143
WBC, median (IQR)			
• Initiation of IV antibiotic	13.7 (9.2–18.4)	14.5 (9.7–20.8)	0.555
• Initiation of PO antibiotic	8.1 (6.2–12.2)	9.1 (7.2–11.7)	0.511
Temperature ≥38 °C, *n* (%)	58 (77.3)	86 (72.3)	0.432
Day of defervescence, median days (IQR)	2 (2–3)	2 (2–3)	0.445
Infectious disease consult, *n* (%)	17 (22.7)	70 (58.8)	<0.001
Blood cultures repeated, *n* (%)	82 (68.9)	26 (34.7)	<0.001
• Positive repeat cultures, *n* (%)	3 (2.5)	0 (0)	0.573
ICU admission, *n* (%)	4 (5.3)	19 (16)	0.038
Vasopressor required, *n* (%)	3 (4)	11 (9.2)	0.255
Empiric regimen, *n* (%)			
• Anti-pseudomonal beta-lactam	5 (6.7)	42 (35.3)	<0.001
• Aztreonam	2 (2.7)	3 (2.5)	
• Ceftriaxone	68 (90.7)	69 (58)	
• Ertapenem	0 (0)	3 (2.5)	
• Fluoroquinolone	0 (0)	2 (1.7)	
Enterobacterales isolated, *n* (%)			
* • Escherichia coli*	58 (77.3)	92 (77.3)	0.995
* • Klebsiella* species	13 (17.3)	21 (17.6)	
* • Proteus mirabilis*	4 (5.3)	6 (5)	
Susceptibility, *n* (%)			
• Aminopenicillin	43 (57.3)	60 (50.4)	0.347
• Cefazolin	75 (100)	41 (91.1)*	0.018
• Ciprofloxacin	73 (97.3)	114 (95.8)	0.708
• TMP/SMX	64 (85.3)	99 (83.2)	0.692
Treatment duration, *n* (%)			
• 7 days	1 (1.3)	12 (10.1)	0.802
• 8–10 days	35 (46.7)	42 (35.3)	
• 11–13 days	19 (25.3)	33 (27.8)	
• 14 days	10 (13.3)	20 (16.8)	
• >14 days	10 (13.2)	12 (10)	

*Standard therapy patients tested for cefazolin susceptibility (*n* = 45) due to change in Clinical Laboratory Standards Institute (CLSI) breakpoint change.

### Outcomes

There was no difference in the rate of mortality or recurrent bacteremia within 30 days between groups (beta-lactam 1.3% vs. standard therapy 1.7%, OR 1.27 [95% CI 0.11–14.2]). One patient (1.3%) who received cephalexin had recurrent *E. coli* bacteremia within 30 days of completing oral treatment. No patients who received beta-lactam therapy expired within 30 days of treatment. In the standard therapy group, two patients (1.7%) who received ciprofloxacin expired within 30 days of therapy. No patients in the standard therapy group had recurrent bacteremia within 30 days.

Fifty-six (74.7%) patients in the beta-lactam group and 87 (73.1%) in the standard treatment group followed up with a primary care physician within 30 days of hospital discharge (*p* = 0.810). Additionally, 8 (10.7%) patients in the beta-lactam group and 14 (11.8%) patients in the standard therapy group were reevaluated in the ED within 30 days (*p* = 0.814) of hospital discharge while 12 (16%) patients in the beta-lactam group and 16 (13.4%) patients in the standard therapy group were re-hospitalized within 30 days (*p* = 0.622). Treatment of recurrent urinary tract infection occurred in 16 (21.3%) patients in the beta-lactam group and 14 (11.8%) patients in the standard treatment group (*p* = 0.073); however, only 5 (6.7%) patients in the beta-lactam group and 6 (5%) patients in the standard therapy group had urinary symptoms documented at follow-up (*p* = 0.510). There were no documented cases of *C. difficile* within 30 days of completing oral antibiotics in either group (*p* = 1.0).

### Predictors of selected oral therapy

Variables included in the multivariate logistic regression model are shown in Table 2. In the final model, ID consultation independently predicted the use of standard oral antibiotic therapy over oral beta-lactam therapy (OR 4.4 [95% CI 2.24–8.62]).

**Table 2.** Variables associated with alternative oral antibiotic use to beta-lactams

Variable	Odds ratio	95% confidence interval
ICU admission	1.67	0.46–6.06
Infectious disease consult	4.4	2.24–8.62
Renal stone	1.07	0.48–2.4
Foley catheter during hospitalization	1.51	0.63–3.66
Foley catheter on discharge	1.05	0.32–3.5
Charlson comorbidity index ≥2	1.55	0.82–2.94

## Discussion

Our findings support definitive oral therapy with cephalexin or amoxicillin dosed at 1 g three times daily for patients with *E. coli, K. pneumonia* or *oxytoca*, and *P. mirabilis* bacteremia from a urinary source. Traditionally, FQ or TMP/SMX have been preferred for definitive oral therapy due to their high bioavailability and ability to achieve serum concentrations similar to IV administration. Due to the safety concerns associated with FQ and TMP/SMX and increasing rates of Enterobacterales resistance, highly bioavailable oral beta-lactam agents administered at high doses provide alternative safe and effective definitive therapy options for bacteremia from a urinary source.

Previous studies evaluating oral beta-lactam therapy for UTI demonstrated increased rates of recurrent infection compared to alternative treatment.^2^ Several studies have evaluated the efficacy of beta-lactams for bacteremia from a urinary source showing promising results for beta-lactams; however, optimal agent, dosing, and duration have not yet been identified.^4–7^ Saad and colleagues conducted a retrospective cohort of 207 patients with *E. coli* bacteremia who received either beta-lactam or FQ as definitive oral therapy.^4^ There was no difference in the primary outcome of clinical cure (beta-lactam, 98% vs. FQ, 94%, *p* = 0.13). Nine different oral beta-lactam regimens were used, including both high- and low-bioavailability agents. Cefixime 400 mg daily and cephalexin 500 mg four times daily were the most frequently utilized oral beta-lactams. Similarly, Sutton and colleagues evaluated 4089 patients with bacteremia from a urinary source; 955 patients received an oral beta-lactam and 3134 patients received either a FQ or TMP/SMX.^5^ The primary outcome of 30-day mortality or recurrent bacteremia occurred in 4.4% of patients in the beta-lactam cohort compared to 3% of patients in the FQ or TMP/SMX cohort (95% CI 0.87–1.95). Eighteen different oral beta-lactam regimens were included. Amoxicillin-clavulanate 875 mg twice daily, cephalexin 500 mg four times daily, and cefpodoxime 200 mg twice daily were the most commonly prescribed oral beta-lactams. More recently, studies by McAlister et al. and Mack et al. also showed no difference in patient outcomes with beta-lactams for Enterobacterales bacteremia from a urinary source compared to TMP/SMX or a FQ.^7,8^ However, these studies still do not address optimal beta-lactam dosing strategies.

Important considerations for definitive oral therapy include bioavailability, protein binding, likelihood of achieving adequate serum concentrations, and patient adherence. Recently, Mponponsuo and colleagues published a propensity-matched cohort study of patients with gram-negative bacteremia who received either a highly bioavailable or less bioavailable oral antibiotic.^17^ Highly bioavailable options included FQ or TMP/SMX while less bioavailable oral antibiotics were beta-lactams. The primary composite outcome of mortality, recurrent bacteremia, and all-cause readmission at 90 days was significantly higher in patients who received a less bioavailable oral antibiotic (17% vs 21.5%; aOR 0.74; 95% CI 0.6–0.93), with recurrent bacteremia driving the difference in the composite primary outcome. This study included multiple sources of infection, with only 50% of patients having a urinary source. Despite these studies having differing outcomes, all the studies utilized a variety of oral beta-lactam regimens and dosing strategies; there were no preferred beta-lactam agents or regimens used for bacteremia. Our health system routinely utilizes ciprofloxacin, TMP/SMX, amoxicillin, and cephalexin as definitive therapy for gram-negative bacteremia from a urinary source. The bioavailability of each of these agents is high at ≥70%.^9–16^ To optimize dosing of beta-lactams, our institution utilizes high-dose amoxicillin 1000 mg three times and cephalexin 1000 mg three times daily. These regimens differ from many of the beta-lactam regimens utilized in previously published retrospective studies and the consensus statement where cephalexin 1000 mg every 6 hours is recommended.^8^ While the serum half-life of cephalexin is approximately one hour, cephalexin achieves high concentrations within the urine with an estimated concentration more than 500 times the MIC breakpoint after a single 500 mg oral dose and concentrations exceeding the breakpoint at 8–12 hours post-dose.^18^ As most patients will have received multiple days of IV antibiotics targeting bacteremia and have negative repeat blood cultures, cephalexin dosed three times daily is optimally aimed to treat the source of infection. Three times daily dosing demonstrated similar patient outcomes and may also improve patient adherence and treatment satisfaction in the outpatient setting compared to more frequent dosing.^19^ Our findings also support that short course IV therapy of 4 days in stable patients prior to switching to definitive oral therapy is safe and effective. This is similar to previously published literature.^4–7^ Importantly, both groups in our study were treated with a median of 11 days of therapy, which supports total durations of therapy less than 14 days for patients treated with oral beta-lactams. This also highlights the need for education when using FQ or TMP/SMX where data for effective courses as short as 7 days of therapy exist.^20^

There are limitations of this study to consider. First, this was a retrospective cohort study of limited size and incidence of the primary outcome. We aimed to overcome these limitations by using a multicenter design that included patients from three institutions with similar dosing recommendations for oral antibiotics when treating bacteremia. These results provide important insight into the efficacy of standard dosing of amoxicillin and cephalexin dosed 1 g three times daily in the treatment of bacteremia and address the limitations of previously published retrospective studies which included a variety of beta-lactam agents and dosing strategies without indicating the appropriateness of dosing based on renal function.^2–7,9^ Second, we relied on accurate documentation within the electronic health record to obtain data. As with all retrospective analyses, this has the ability to introduce selection bias; however, our patients were well-matched at baseline with regard to patient and treatment characteristics. The frequency of the primary outcome in our study was rare; therefore, we were unable to complete regression analysis to control for potential confounders between groups. We did perform logistic regression to eliminate confounders in identifying factors independently associated with FQ or TMP/SMX prescribing. The only variable that was a significant determinant in favoring FQ or TMP/SMX prescribing was ID consultation. We hypothesize that our ID physicians may be more comfortable using these agents despite the safety concerns in patients with complicated infections, such as bacteremia. Finally, patients with structurally complicated infections that would require prolonged courses of antibiotics including renal abscesses or lobar nephronia were excluded; therefore, the effectiveness of oral beta-lactams for definitive therapy in these more complicated infections with structural abnormalities remains unknown. We did, however, include complex patients with a urinary catheter present on admission and those with renal stones or obstruction requiring operative intervention which supports oral beta-lactam therapy in these patient populations.

Patients treated with high-dose oral beta-lactams for gram-negative bacteremia from a urinary source had similar outcomes to those treated with FQ or TMP/SMX. Cephalexin and amoxicillin dosed 1 g three times daily may provide a safe alternative to FQ or TMP/SMX for some patients and three times per day dosing may improve adherence over more frequent dosing regimens. Treatment durations of less than 14 days were safe and efficacious across oral treatment strategies.

## Supporting information

Geyer et al. supplementary material Geyer et al. supplementary material

## Supplementary material

For supplementary material accompanying this paper visit http://doi.org/10.1017/ash.2023.435. click here to view supplementary material
