# Supplementary material for: Outcomes of high-dose oral beta-lactam definitive therapy compared to fluoroquinolone or trimethoprim-sulfamethoxazole oral therapy for bacteremia secondary to a urinary tract infection
Source: Antimicrob Steward Healthc Epidemiol. 2023 Sep 8;3(1):e148. doi: 10.1017/ash.2023.435 (PMC10523554; doi:10.1017/ash.2023.435)
Supplement: Geyer et al. supplementary material [file S2732494X23004357sup001.docx]

**Supplementary Table 1: Bioavailability of Oral Antibiotics**

| **Penicillins** | **Bioavailability** | **Protein binding** | **Urinary excretion** |
| --- | --- | --- | --- |
| Amoxicillin | 70-80% ^9, 10^ | 17-20% ^9^ | 60% ^21^ |
| Amoxicillin/clavulanate | 70-80% ^9, 10^ | 18-25% ^9^ | 25-40% ^22^ |
| **Cephalosporins** | |  |  |
| Cephalexin | 95% ^11^ | 10-19% ^9^ | 90% ^9^ |
| Cefadroxil | 90% ^12^ | 20% ^12^ | 90% ^12^ |
| Cefuroxime | 30-52% (increased with food) ^13^ | 33-50% ^13^ | 66-100% ^13^ |
| Cefpodoxime | 29-53% (increased with food) ^9^ | 18-30 ^9^ | 29-33% ^23^ |
| Cefdinir | 21-25% ^14^ | 60-70% ^14^ | 12-18% ^14^ |
| **Fluoroquinolones** | |  |  |
| Ciprofloxacin | 70% ^15^ | 20-40% ^15^ | 35-70% ^24^ |
| Levofloxacin | 99% ^16^ | 24-38% ^16^ | 87% ^25^ |
| **Other** | |  |  |
| Sulfamethoxazole/trimethoprim | 100% ^9^ | SMX: 70% ^9^  TMP: 44% ^9^ | SMX: 84.5% ^26^  TMP: 66.8% ^26^ |

**Supplementary Table 2: Oral Antibiotic Dosing Reference for Renal Function (Creatinine Clearance)**

| Oral Antibiotic | ≥ 50 mL/min | 30-49 mL/min | 10-29 mL/min | < 10 mL/min or HD |
| --- | --- | --- | --- | --- |
| Amoxicillin | 1000 mg TID | 1000 mg TID | 1000 mg BID | 1000 mg BID |
| Cephalexin | 1000 mg TID | 1000 mg TID | 1000 mg BID | 1000 mg BID |
| Ciprofloxacin | 500-750 mg BID | 500-750 mg BID | 500-750 mg daily | 500 mg daily |
| Levofloxacin | 500-750 mg daily | 750 mg x 1  then 500 mg q48h | 750 mg x 1  then 500 mg q48h | 500 mg q48h |
| TMP/SMX | 2 DS BID | 2 DS BID | 1-2 DS BID | 2 DS daily |
